# Lp(a) in the Horizon of Diagnostics and Therapy

**DOI:** 10.3390/ijms27010290

**Published:** 2025-12-27

**Authors:** Pietro Formisano, Elena Vianello, Elena Dozio, Lorenza Tacchini, Luigina Romani, Luigi Frati, Francesco Curcio, Marina Maria Bellet, Massimiliano Marco Corsi-Romanelli

**Affiliations:** 1 Department of Translational Medicine, Federico II University of Naples, 80131 Naples, Italy; 2 Department of Biomedical Sciences for Health, University of Milan, 20133 Milan, Italy; 3 Experimental Laboratory for Research on Organ Damage Biomarkers, IRCCS Istituto Auxologico Italiano, 20149 Milan, Italy; 4 Department of Experimental Medicine and Biochemical Sciences, University of Perugia, 06132 Perugia, Italy; 5 Istituto di Ricovero e Cura a Carattere Scientifico (IRCCS) Neuromed, 86077 Pozzilli, Italy; 6 Department of Medicine, University of Udine, 33100 Udine, Italy; 7 Laboratory Medicine Department, Azienda Sanitaria Universitaria Friuli Centrale, 33100 Udine, Italy; 8 Department of Medicine and Surgery, University of Perugia, P.le L. Severi 1, 06132 Perugia, Italy; 9 Department of Experimental and Clinical Pathology, IRCCS Istituto Auxologico Italiano, 20149 Milan, Italy

**Keywords:** Lp(a), cardiovascular risk, therapy

## Abstract

Low-density lipoprotein cholesterol (LDL-C) has traditionally been the primary biomarker used to assess cardiovascular risk. However, a substantial proportion of cardiovascular events occur in individuals with LDL-C levels within the normal range, highlighting the need for additional risk markers. Lipoprotein(a) [Lp(a)] has emerged as an independent and genetically determined cardiovascular risk factor that is not adequately captured by conventional lipid profiling. Elevated Lp(a) levels are associated with an increased risk of atherosclerotic cardiovascular disease, including coronary artery disease, ischemic stroke, and calcific aortic valve stenosis, and appear to be particularly relevant in the context of premature cardiovascular events. The pathogenicity of Lp(a) is driven by distinct mechanisms that extend beyond cholesterol transport. These include pro-atherogenic, pro-inflammatory, and pro-thrombotic effects mediated largely by oxidized phospholipids carried by the particle and by the structural properties of apolipoprotein(a), which interfere with fibrinolysis. Despite its strong and stable genetic determination, Lp(a) remains underrecognized and inconsistently measured in clinical practice, partly due to historical limitations in assay standardization and reporting. This minireview summarizes current knowledge on the pathophysiological mechanisms underlying elevated Lp(a), discusses its clinical implications for cardiovascular risk assessment, and highlights the importance of standardized Lp(a) measurement in routine practice, particularly in light of emerging Lp(a)-targeted therapies.

## 1. Introduction

Lipoprotein (a), usually abbreviated as Lp(a), is commonly referred to as “Lipoprotein little a.” Lp(a) bears a resemblance to low-density lipoprotein (LDL), except it contains an additional protein called “apolipoprotein A”(ApoA) [1,2]. Like LDL, which is a cholesterol-rich lipoprotein, Lp(a) causes plaque formation in the arteries. The ApoA confers upon Lp(a) an additional characteristic, allowing it to display prothrombotic properties via an antifibrinolytic mechanism predominantly driven by ApoA. The combination of accelerated atherosclerotic progression and a prothrombotic effect results in a markedly increased risk of cardiovascular events [2,3]. Interestingly, Lp(a) is considered as an independent risk factor for cardiovascular disease (CVD) since its level in the bloodstream is solely determined by its own genetic make-up. Indeed, unlike other cardiac risk factors, Lp(a) cannot be modified through dietary changes or physical exercise. Lp(a) levels remain stable throughout life, with comparable values observed at ages 5 and 45 [4]. The sole exception is that it can increase during menopause and due to certain medical conditions such as chronic kidney disease [4]. Research has indicated that between one-third and one-half of individuals with familial hypercholesterolemia (FH) also exhibit elevated Lp(a) levels [2]. This places them at a heightened risk for earlier onset of heart disease or stroke compared to individuals with FH alone. Consequently, more intensive management of additional cardiovascular risk factors may be warranted. Moreover, in individuals with elevated Lp(a) levels, screening of biological relatives may be considered. Numerous expert organizations, including the National Lipid Association (NLA), the American Heart Association (AHA), and the American College of Cardiology (ACC), concur that a level exceeding 50 mg/dL or 125 nmol/L should be considered a cardiovascular risk-enhancing factor [5]. It is imperative to note that Lp(a) can be measured in both mg/dL and nmol/L, necessitating the verification of the units of measurement to ascertain whether levels are elevated (2).

Accurate quantification of Lp(a) is essential for evaluating cardiovascular risk, yet its measurement poses significant analytical challenges owing to the marked structural heterogeneity of ApoA. The variability in the number of kringle IV type-2 repeats leads to ApoA isoforms with different molecular sizes, which interact differentially with antibodies used in immunoassays. Therefore, early and isoform-sensitive methods yielded considerable discrepancies between laboratories, complicating comparisons across studies and limiting clinical interpretation [6,7]. These issues prompted international efforts to standardize Lp(a) detection, culminating in the recommendation to adopt isoform-insensitive assays calibrated to the World Health Organization/International Federation of Clinical Chemistry (WHO/IFCC) reference material. This standard allows reporting in nmol/L, reflecting particle number rather than mass and minimizing the influence of ApoA size variability [8]. The continued use of mg/dL in some laboratories introduces inconsistencies because mass-based values do not proportionally correlate with particle count, particularly in individuals with large ApoA isoforms.

Despite improved standardization, several technical limitations remain. Not all commercially available assays fully eliminate isoform-dependent bias, and calibration procedures vary among manufacturers, leading to subtle but clinically relevant differences in measured concentrations [9]. Furthermore, pre-analytical factors—such as sample stability, storage temperature, and potential interference from lipemia or hemolysis—can affect assay performance. The absence of universal cut-off values across international guidelines further complicates the adoption of uniform clinical thresholds. Emerging technologies, such as mass spectrometry-based methods or advanced nephelometric assays, hold promise for improving precision, but these approaches are not yet widely implemented in routine clinical settings due to cost, complexity, and limited standardization [10]. As novel Lp(a)-lowering therapies progress through advanced clinical trials, the need for globally harmonized and highly accurate Lp(a) measurement becomes increasingly critical. Reliable quantification will be essential for patient selection, therapeutic monitoring, and post-treatment risk assessment. Continued refinement of assay technologies, broader implementation of standardized calibration materials, and international agreement on reference intervals will be fundamental steps toward integrating Lp(a) more effectively into clinical practice and research frameworks.

## 2. The Dual Role and Pathophysiology of Lp(a) in Cardiovascular Risk

Lp(a) is a distinct plasma lipoprotein particle, recognized as a causal, genetically determined, and independent risk factor for the development of atherosclerotic cardiovascular disease (ASCVD) [11]. Although its precise physiological role largely remains a subject of research, with hypotheses suggesting a function in oxidized phospholipid (OxPLs) transport or tissue repair, its pathological impact at elevated plasma concentrations is unequivocal [12]. Lp(a)’s unique structure is the basis of its pathogenicity: it consists of an LDL-like core (rich in cholesterol) and an additional protein, ApoA, which is covalently linked to the LDL’s apolipoprotein B-100 (ApoB-100) via a disulfide bridge. ApoA is highly polymorphic and, crucially, exhibits significant structural homology to plasminogen, the key element of fibrinolysis, thus conferring upon Lp(a) a dual threat: atherogenesis and thrombogenesis [13].

The contribution of Lp(a) to atherogenesis manifests through several mechanisms starting with arterial wall infiltration. Due to its similarity to LDL, Lp(a) easily accumulates in the subendothelial space, where it is retained by the extracellular matrix and deposits cholesterol, promoting atherosclerotic plaque formation. Even more critical is its role as the primary carrier of oxidized phospholipids (OxPLs). These potent molecules trigger a pro-inflammatory and pro-oxidative response, which is essential for disease progression. Once retained in the vascular wall, OxPLs contribute to endothelial dysfunction and stimulate the recruitment and transformation of macrophages into foam cells, accelerating plaque evolution. Lp(a) is also implicated in pro-calcific processes, contributing to the calcification of the aortic valve and coronary arteries, an additional independent risk factor [14,15]. Although the vascular toxicity associated with high Lp(a) levels is well documented, its physiological function remains incompletely defined. One of the leading hypotheses suggests that Lp(a) may have evolved to facilitate the transport and clearance of oxidized phospholipids, thereby acting as a scavenger under conditions of oxidative stress [16]. Another proposed role relates to tissue repair, as the structural similarity between apolipoprotein(a) and plasminogen supports the idea that Lp(a) could influence extracellular matrix remodeling or wound-healing responses, potentially participating in fibrinolysis-independent pathways of tissue restoration [17,18]. Additional putative roles include modulation of immune processes and maintenance of endothelial barrier function, although these remain speculative due to limited mechanistic validation. This uncertainty reflects an important gap in current knowledge and highlights the need for further studies to delineate whether any physiological benefits coexist with the well-established pathogenic effects of Lp(a).

In parallel, Lp(a) exerts a powerful pro-thrombotic effect through the action of its Apo(a) component [19]. Given the notable structural similarity between ApoA and plasminogen, Lp(a) acts as a competitive inhibitor of the fibrinolytic system. ApoA actively competes with plasminogen for binding sites on fibrin (the main constituent of the clot) and on endothelial cells. By occupying these sites, Lp(a) reduces the efficiency with which true plasminogen can be activated to plasmin, the enzyme responsible for fibrinolysis (clot dissolution). This antifibrinolytic effect compromises the body’s natural ability to dissolve existing clots, resulting in increased thrombus stability and persistence. This hemostatic imbalance significantly increases the risk of acute thrombotic events, such as myocardial infarction and ischemic stroke, particularly in the presence of vulnerable atherosclerotic plaques [20].

The most distinctive aspect of Lp(a) lies in its genetic control. Plasma Lp(a) levels are highly heritable, with over 90% of the variability determined by the *LPA* gene [21]. This genetically predetermined nature implies that, unlike LDL cholesterol (LDL-C), Lp(a) levels remain largely constant throughout life and are non-modifiable by conventional lifestyle interventions, such as diet and exercise. The only exceptions to this constancy are specific physiological or pathological conditions like menopause and chronic kidney disease (CKD). Clinically, elevated Lp(a) (generally defined as above 50 mg/dL or 125 nmol/L) [21] is a risk factor that requires particular attention, especially when coexisting with FH, where the combination of the two genetic conditions exponentially increases the risk of premature and severe ASCVD [22].

## 3. Lp(a) Clinical Use

The Lp(a) test should be requested by a specialist primarily for patients with risk factors, such as a personal or family history of premature heart attack or stroke, a high estimated 10-year cardiovascular risk, and younger individuals with FH. Pediatric guidelines also recommend measuring Lp(a) as part of cardiovascular risk assessment, as Lp(a) measurement not only improves prediction of cardiovascular risk but also enhances diagnostic accuracy for familial hypercholesterolemia when LDL-C is corrected for Lp(a)-cholesterol. Evidence further supports measuring Lp(a) in children suspected of FH, given that DNA analysis together with Lp(a) assessment can help differentiate those with definite FH from those without an FH mutation but with elevated Lp(a), particularly in the absence of validated diagnostic tools for FH in pediatric populations [5,23,24]. Recent guidelines increasingly recommend measuring Lp(a) levels at least once in a person’s life. Lp(a) levels are genetically determined and do not significantly change over time, with the final concentration usually observed at the age of 5 years [9,25]. It is estimated that between 10 and 20% of the general population may have high levels. Additionally, the values vary significantly between individuals, making it challenging to establish a single reference point. Values below 10 mg/dL are considered normal, values between 30 and 50 mg/dL are borderline, and values over 50 mg/dL should be considered a cardiovascular risk-enhancing factor [5,26]. Like LDL, Lp(a) is considered a risk factor for cardiovascular disease, but it does not respond to standard LDL-lowering therapies, such as diet, exercise, or statin therapy [25]. An increasing number of cardiologists advocate for more widespread Lp(a) testing [26]. For instance, Dr. Cannon now measures it in most patients who have or are at high risk of cardiovascular disease. Interestingly, individuals with high Lp(a) levels often have close family members who experienced heart attacks or strokes in their 50s or even earlier [25,27]. Despite lacking traditional risk factors such as high LDL cholesterol, diabetes, or high blood pressure, some of these individuals appear to be unlikely candidates for developing heart disease. Many individuals have one or more risk factors, and elevated Lp(a) contributes to their overall susceptibility to cardiovascular issues. We consider high Lp(a) a risk factor that may require more intensive LDL lowering, along with maintaining a healthy lifestyle [27]. This includes adopting a predominantly plant-based diet, engaging in regular physical activity, ensuring adequate sleep, and effectively managing stress. In selected individuals with elevated Lp(a) levels, intensive LDL-cholesterol-lowering strategies may contribute to further cardiovascular risk reduction. The role of low-dose acetylsalicylic acid in this setting remains uncertain. While the prothrombotic properties associated with elevated Lp(a) provide a biological rationale for its use, current clinical guidelines do not recommend routine acetylsalicylic acid therapy for primary prevention because of the limited net cardiovascular benefit and the increased risk of bleeding. Available evidence in individuals with elevated Lp(a) is derived mainly from observational studies, and randomized controlled trials specifically targeting this population are currently lacking [28,29].

Individuals with elevated Lp(a) should consult a cardiologist for personalized advice and should also recommend Lp(a) testing for their close family members [2].

In Europe and Canada, current clinical guidelines recommend that all individuals undergo Lp(a) measurement at least once in their lifetime. This recommendation is explicitly included in the 2019 ESC/EAS dyslipidemia guidelines and in the 2021 Canadian Cardiovascular Society (CCS) dyslipidemia recommendations [5,30]. In contrast, U.S. guidelines adopt a more selective approach. The ACC/AHA and the NLA advise Lp(a) testing primarily in individuals with a personal history of premature cardiovascular disease—defined as myocardial infarction, stroke, peripheral artery disease, or aortic stenosis occurring before 55 years of age in men or 65 years in women—as well as in those with a first-degree relative affected by premature cardiovascular disease [31,32]. Testing is also recommended for individuals with markedly elevated LDL-cholesterol concentrations (≥190 mg/dL) and for first-degree relatives of patients with known elevated Lp(a) levels [32]. A deeper understanding of the clinical utility of Lp(a) emerges when its associated disease manifestations are compared with those predicted by existing cardiovascular biomarkers. Traditional lipid parameters such as LDL-cholesterol (LDL-C), non-HDL-cholesterol, and ApoB are central to assessing atherogenic lipoprotein burden; however, they do not capture the distinct pathogenic pathways driven by Lp(a). Elevated Lp(a) is strongly associated with premature coronary artery disease, recurrent myocardial infarction, ischemic stroke, and calcific aortic valve stenosis, conditions that may occur even when LDL-C and ApoB are within guideline-recommended ranges [33,34]. This discordance reflects the unique properties of Lp(a), including its high content of oxidized phospholipids, its inflammatory potential, and its pro-thrombotic activity mediated by the Apo(a) structural homology to plasminogen. Compared with ApoB—which quantifies the overall number of atherogenic particles—Lp(a) identifies a genetically defined subgroup at disproportionately high risk relative to their total particle burden [35]. Likewise, LDL particle number (LDL-P) correlates with plaque development but does not capture the valvular calcification or thrombotic tendencies uniquely associated with Lp(a). Inflammatory biomarkers such as high-sensitivity C-reactive protein (hs-CRP) reflect systemic inflammation but do not identify individuals with inherited Lp(a)-mediated vascular risk [36]. Structural imaging markers, including coronary artery calcium (CAC) and carotid intima–media thickness (cIMT)**,** indicate cumulative disease burden; Lp(a), in contrast, provides information about early predisposition, often long before structural abnormalities are detectable [37]. Emerging biomarkers further highlight the complementary value of Lp(a). Lipoprotein-associated phospholipase A2 (Lp-PLA2) and myeloperoxidase (MPO) quantify vascular inflammation and oxidative stress but do not elucidate the genetic risk component carried by Lp(a). Similarly, high-sensitivity cardiac troponins, although powerful predictors of incident cardiovascular events, signal myocardial injury rather than inherited susceptibility [38]. When interpreted together with these biomarkers, Lp(a) enriches risk stratification by identifying patients whose cardiovascular phenotype is driven by mechanisms not detectable through lipid load, inflammatory markers, or structural imaging alone. Overall, integrating Lp(a) measurement with established and emerging biomarkers enables a more nuanced characterization of cardiovascular risk. This multimodal approach helps identify individuals whose clinical manifestations—such as early-onset ASCVD or disproportionate progression of valvular calcification—are not adequately explained by traditional metrics, thereby informing more personalized prevention and management strategies.

## 4. How to Lower Lp(a)?

Current evidence indicates that lifestyle measures and the management of coexisting conditions exert only minimal effects on circulating Lp(a), whose plasma concentration is largely determined by inherited variation in the *LPA* gene. The strong genetic influence reflects the unique molecular architecture of Apo(a), which is composed of multiple kringle domains, particularly the highly polymorphic kringle IV type 2 (KIV-2) repeats. The number of KIV-2 copies varies extensively among individuals and is inversely correlated with circulating Lp(a) concentrations, as smaller *LPA* isoforms with fewer KIV-2 repeats are synthesized and secreted more efficiently by hepatocytes. ApoA is produced exclusively in the liver, where its biosynthesis involves translation of the *LPA* mRNA, post-translational glycosylation within the endoplasmic reticulum, and further processing along the secretory pathway before ApoA is released into the extracellular space. Circulating Lp(a) particles form through a non-covalent, followed by covalent, interaction between ApoA and ApoB on LDL-like particles, a process thought to occur predominantly at the hepatocyte surface. Dietary modifications, including the substitution of saturated fats with other macronutrients, induce only modest changes in Lp(a)—typically increases of 10–15%—consistent with the limited environmental influence on ApoA biosynthesis and secretion [39]. Hormonal factors exert slightly greater effects: androgen or estrogen replacement therapies can alter hepatic expression or processing of ApoA, whereas thyroid dysfunction induces only minor fluctuations [39]. Comorbidities such as chronic kidney disease, nephrotic syndrome, and hepatic impairment may interfere with apo(a) production or clearance; advanced liver disease often results in reduced Lp(a) due to diminished hepatocellular synthetic capacity [39]. Therapeutic strategies capable of producing substantial reductions in Lp(a) remain limited. Although niacin and estrogen-based therapies can decrease Lp(a), these effects have not been accompanied by demonstrable cardiovascular benefit in randomized trials [11]. Conventional lipid-lowering therapies such as statins do not meaningfully influence ApoA production or the assembly of Lp(a), whereas proprotein convertase subtilisin/kexin type 9 (PCSK9) inhibitors provide only modest reductions and are not approved specifically for Lp(a)-lowering indications [11]. Lipoprotein apheresis remains the only established intervention capable of acutely reducing both Lp(a) and LDL-C concentrations, although its invasive nature and restricted availability limit its clinical applicability to patients at extremely high cardiovascular risk [40]. Persistent cardiovascular risk despite intensive LDL lowering is increasingly recognized as partly attributable to elevated Lp(a), reflecting the proatherogenic, proinflammatory, and prothrombotic molecular properties conferred by the apo(a) moiety [41]. Major advances have emerged with the development of molecular therapies designed to inhibit ApoA synthesis at its source. Antisense oligonucleotides and small interfering RNA molecules—including pelacarsen, olpasiran, and SLN360—target *LPA* mRNA in hepatocytes, thereby reducing translation of ApoA and preventing subsequent Lp(a) assembly. These agents have produced reductions in circulating Lp(a) of 70–100% in early clinical studies, with generally mild and transient adverse events [42,43]. The phase 3 Lp(a) HORIZON trial is expected to determine whether substantial suppression of apo(a) biosynthesis translates into lower rates of cardiovascular events [39]. In parallel, small-molecule inhibitors such as muvalaplin act through a different molecular mechanism, preventing the interaction between ApoA and ApoB100 and thereby blocking the extracellular assembly of Lp(a). In phase 2 studies, muvalaplin reduced Lp(a) by up to ~86% with favorable tolerability [40,44,45]. Another promising agent, zerlasiran—an siRNA therapeutic—suppresses hepatic *LPA* expression and has demonstrated sustained reductions exceeding 80% in individuals with atherosclerotic cardiovascular disease [44]. Collectively, these molecularly targeted therapies represent a rapidly evolving field with the potential to provide the first effective pharmacological approaches for reducing Lp(a)-mediated cardiovascular risk, pending confirmation of long-term efficacy and safety.

## 5. Overview of Genetic Testing for Lp(a)

Plasma concentrations of Lp(a) are determined predominantly by inherited variation within the *LPA* gene, as previously mentioned in this review, which encodes Apo(a). A major source of interindividual variability is the number of kringle IV type-2 (KIV-2) repeats, a structural polymorphism that generates apo(a) isoforms of markedly different sizes. Smaller isoforms, typically associated with fewer KIV-2 repeats, correlate with higher circulating Lp(a) levels, whereas larger isoforms are linked to lower concentrations [46,47,48]. The heritability of Lp(a) exceeds 80–90%, among the highest reported for lipid traits [49,50]. In addition to the KIV-2 copy-number variation, specific *LPA* single-nucleotide variants—such as rs3798220 and rs10455872—also influence Lp(a) concentrations and contribute to cardiovascular risk [51,52].

Despite the strong genetic regulation of Lp(a), current clinical guidelines do not support targeted genetic testing for individuals with elevated Lp(a). Major societies, including the ESC, the NLA, and the AHA/ACC, consistently recommend direct plasma Lp(a) measurement as the appropriate clinical tool for assessing Lp(a)-related cardiovascular risk [5,32,53].

Although major guidelines recommend measuring Lp(a) at least once in adults to clarify cardiovascular risk, routine LPA sequencing is not currently advised as there are no validated criteria to classify the pathogenicity of LPA variants and no evidence that genotyping provides clinically actionable information beyond plasma Lp(a) measurement.

Increasing interest has been directed toward polygenic risk scores (PRS) incorporating multiple genetic variants associated with Lp(a) levels or cardiovascular outcomes. Preliminary studies suggest that Lp(a)-related PRS may offer additional refinement in cardiovascular risk stratification when combined with traditional factors [54,55,56]. Nonetheless, these tools are still under investigation, lack robust validation in diverse cohorts, and are not endorsed by current guidelines for routine clinical use.

In summary, although Lp(a) levels are predominantly determined by genetic factors, genetic testing does not presently play a role in the routine evaluation or management of patients with elevated Lp(a). Direct measurement of circulating Lp(a) remains the only guideline-supported method for assessing Lp(a)-associated cardiovascular risk. Future research will clarify whether broader *LPA* genotyping or Lp(a)-specific PRS will provide clinical value, particularly as novel Lp(a)-lowering therapies progress through late-stage development.

## 6. Conclusions

Elevated Lp(a) levels represent an important and independent risk factor for cardiovascular disease. Lp(a) concentrations are largely genetically determined, with heritability estimates ranging from 70% to 90%, and tend to remain stable throughout life. It is estimated that up to 20% of the global population has elevated Lp(a) levels, which are associated with an increased risk of myocardial infarction and other major adverse cardiovascular events. Despite their strong genetic basis and well-established association with cardiovascular risk, elevated Lp(a) levels often remain underrecognized in clinical practice.

Unlike other risk factors, Lp(a) is not well understood, and current tests lack standardization. Due to variations in Lp(a) particle size, commonly employed laboratory tests are insufficient. Physicians and researchers in this field advocate for the development of more precise, standardized tests that measure Lp(a) levels in nanomoles per liter rather than milligrams per deciliter. All the aspects reviewed in this article are summarized in Table 1. By enhancing our comprehension of Lp(a) and refining testing methodologies, we can better assist physicians in identifying high-risk patients. This progress will also support research into the mechanisms linking Lp(a) to heart disease.

## Figures and Tables

**Table 1:** Overall, Knowledge of Lp(a). This table summarizes the content of the review, illustrating the current understanding of Lp(a), including the pathophysiological mechanisms in which this protein is involved, its reference values expressed as risk thresholds recommended by the main international guideline published by ESC Clinical Practice Guidelines(2025), the epidemiology regarding its prevalence in the population, and the clinical conditions in which Lp(a) is upregulated. Within this clinical context, we also examine current therapeutic approaches aimed at reducing Lp(a) levels, with particular attention to emerging drug therapies.

Pathophysiological Mechanisms	-Pro-Atherogenic: Cholesterol and Oxidized Deposition -Pro-Inflammatory: Trasport of Oxidized Phospholipids (OxPL) -Pro-Thrombotic: Interference with Plasminogen and Reduced Fibrinolysis	[9,27]
Cardiovascular risk-enhancing factor	-<30 mg/dL (<75 nmol/L): low risk −30–50 mg/dL (75–125 nmol/L): intermediate risk -≥50 mg/dL (≥125 nmol/L): high risk	[5,14,53]
Epidemiology	−20–25% of the population has elevated levels -Strong genetic determination (80–90%)	[4]
Associated Condition	-Coronary artery disease -Calcific aortic valve stenosis -Ischemic stroke	[4,10]
Current Therapies	-Statin, fibrates, ezetimibe: minimal effect. -Niacin: 20–40% reduction, not recommended -Lipoprotein apheresis: selected cases. -PCSK9 inhibitors: ~20–30% reduction in Lp(a); cardiovascular benefit primarily driven by LDL-C lowering	[4,9]
Emerging Therapies	-ASO (Pelacarsen): up to 80% reduction. -siRNA (Olpasiran, Lepodisiran, Zerlasiran): >80–90% reduction -Small molecules (Muvalaplin):in development.	[23,26]

## Data Availability

No new data were created or analyzed in this study. Data sharing is not applicable to this article.

## Abbreviations

The following abbreviations are used in this manuscript:

ACC	American College of Cardiology
AHA	American Heart Association
ApoA	Apolipoprotein(a)
ApoB	Apolipoprotein(b)
ASCVD	Atherosclerotic Cardiovascular Disease
CAC	Coronary artery calcium
cIMT	Carotid intima–media thickness
CKD	Chronic kidney disease
CVD	Cardiovascular disease
ESC/EAS	European Society of Cardiology/European Atherosclerosis Society
FH	Familial hypercholesterolemia
hs-CRP	high-sensitivity C-reactive protein
KIV-2	Kringle IV type 2
LDL	Low density lipoprotein
LDL-C	LDL cholesterol
LDL-P	LDL particle number
Lp(a)	Lipoprotein a
Lp-PLA2	Lipoprotein-associated phospholipase A2
MPO	Myeloperoxidase
NLA	National Lipid Association
OxPLs	Oxidized phospholipid
PCSK9	Proprotein convertase subtilisin/kexin type 9
PRS	Polygenic risk scores (PRS)
WHO/IFCC	World Health Organization/International Federation of Clinical Chemistry
